# Gender and maladaptive personality correlates in problem gambling and over-indebtedness: Novel findings from a cross-sectional study in Sweden

**DOI:** 10.1016/j.heliyon.2023.e18844

**Published:** 2023-08-01

**Authors:** Nikoleta Komzia, Martin Bäckström, Anders Håkansson

**Affiliations:** a Department of Psychology, Faculty of Social Sciences, Lund University, Lund, Sweden; b Department of Clinical Sciences Lund, Psychiatry, Faculty of Medicine, Lund University, Lund, Sweden; c Gambling Disorder Unit, Malmö Addiction Center, Malmö, Sweden

**Keywords:** problem gambling, maladaptive personality traits, over-indebtedness, PID-5-BF, PGSI

## Abstract

Although most individuals consider gambling to be an innocent and fun activity, when it develops into problem gambling, it can have detrimental outcomes to one's life, such as over-indebtedness. This cross-sectional study explores the role of maladaptive personality traits and gender in both problem gambling and over-indebtedness, in an online sample of 1479 adult gamblers (65% males) in Sweden. Participants were administered the Problem Gambling Severity Index (PGSI), the Personality Inventory for DSM-5-Brief Form (PID-5-BF), and questions addressing subjective over-indebtedness and other risk factors. Quasi-Poisson loglinear models and logistic regression analyses demonstrated that Disinhibition (*OR* = 1.38, 95% CI [1.24, 1.53]), and Antagonism (*OR* = 1.23, 95% CI [1.14, 1.34]) showed the strongest associations to problem gambling, and that only Disinhibition (*OR* = 1.72, 95% CI [1.22, 1.43]) and Antagonism (*OR* = 2.00, 95% CI [1.52, 2.66]) were significantly related to over-indebtedness. The prevalence of problem gambling and over-indebtedness was more common among women, and gender moderated the univariate relationships of Negative Affectivity, Disinhibition and Psychoticism to problem gambling. These findings call for future research addressing maladaptive personality traits, problem gambling and over-indebtedness, and highlight the need for tailored interventions and prevention strategies, particularly for women who may be at higher risk.

## Highlights

• Problem gambling and over-indebtedness were more common among women than men.
• Disinhibition and Antagonism showed the strongest associations with problem gambling.
• Only Disinhibition and Antagonism were uniquely related to over-indebtedness.
• Gender moderated the univariate relationships of Negative Affectivity, Disinhibition and Psychoticism to problem gambling.

## 1 Introduction

Problem gambling is typically defined as problematic gambling behavior that does not constitute a gambling disorder (GD) diagnosis. Problem gambling has a prevalence estimate of 1.29% while moderate or at-risk gambling has a prevalence estimate of 2.43%, according to a meta-analytic review from 2022 [1]. Research has consistently shown a higher prevalence of problematic gambling among men than women [2], both in the general population [[3], [4], [5], [6]], and clinical contexts [[7], [8], [9]]. While this has been observed across Europe [5,[10], [11], [12], [13], [14], [15], [16]], including Sweden [3], new studies indicate that Swedish women, compared to men, are more prone to develop problem gambling and having worse financial outcomes [17,18]. Gender differences in personality traits may explain these new results, but gender comparisons are scarce, possibly due to male overrepresentation in past research [8]. Considering the new findings, research on personality factors could help differentiate between genders and promote better clinical diagnoses.

But is there a relationship between personality traits and problem gambling? There are ample studies employing several models, dedicated to answering this question. For instance, researchers have used the Five-Factor Model (FFM) of personality, which describes individual differences based on five main dimensions: Neuroticism, Extraversion, Openness, Agreeableness, and Conscientiousness [19]. Problem gamblers, when compared to healthy controls, have been reported to rate higher on Neuroticism [20,21], while it has been shown that GD is also associated with low ratings of Openness [21] and Agreeableness [[22], [23], [24], [25]].

Another body of research has investigated and supported the presence of impulsivity and sensation-seeking in problematic gambling behavior [[26], [27], [28], [29], [30], [31]]. Even though sensation-seeking can be seen in individuals without gambling-related hardship [20], findings suggest that problem gamblers score higher on the sensation-seeking subscales of disinhibition and boredom susceptibility [32]. Nonetheless, the results on sensation-seeking tend to differ. For instance, when compared to the general population, pathological gamblers were found to have higher rates of psychoticism, neuroticism, state and trait anxiety scores but significantly lower scores of sensation-seeking [33]. Additionally, earlier research from various countries and cultures has revealed that sensation-seeking is related to extraversion and psychoticism [34,35], which are traits typically associated with gambling involvement [36,37].

Recently there has been a focus on the role of maladaptive personality, and in particular, the Alternative DSM-5 Model for Personality Disorders [38], which has been developed to better capture the maladaptive or extreme ends of the common FFM personality traits [19]. This model replaces Neuroticism with Negative Affectivity (negative emotions and behaviors, such as dependence or self-harm), Extraversion with Detachment (avoidance of socio-emotional experiences and relationships), Agreeableness with Antagonism (opposition to others, egocentrism and lack of empathy), Conscientiousness with Disinhibition (searching fast gratification and impulsive behaviors) and Openness with Psychoticism (strange or unusual behaviors and thoughts). For the assessment of these traits, the Personality Inventory for DSM-5 (PID-5) [39] was developed, which shows many similarities with the FFM [19], as the dimensions have a similar structure [40].

Studies from adolescent populations suggest that Antagonism, Disinhibition [41,42], and Negative Affectivity [43] predict gambling involvement. Studies in adult populations suggest that Disinhibition is linked to the decision to chase losses, and that Disinhibition, Detachment, and Psychoticism can predict one's persistence in chasing losses [44]. Moreover, GD has been connected to Detachment and Antagonism [45], while a link has also been found between GD and the domains of Disinhibition, Detachment, and Psychoticism [46]. Although most maladaptive personality traits have been associated with problematic gambling, the weakest support has been found for Antagonism and Negative Affectivity [47,48].

One of the most common and severe outcomes of problematic and disordered gambling is financial debt [49]. On the extreme end is over-indebtedness, which typically suggests difficulties to meet debt payment obligations, and has both been linked with depressive symptoms and sleeping difficulties [50]. With regards to personality traits, it has been shown that individuals with low levels of dutifulness or conscientiousness [51] and a propensity towards neuroticism and feelings of guilt [52] are more likely to experience over-indebtedness. Impulsivity has also been linked to both indebtedness behavior [53] and over-indebtedness [54]. However, as these relationships have not yet been studied in a population of problem gamblers, and research on maladaptive personality traits and over-indebtedness is largely missing, it would be worthwhile to examine whether problem gamblers with specific personality traits are at a greater risk of being over-indebted compared to others.

The role of personality traits in problem gambling has been widely researched, but traits on the maladaptive end have not been extensively explored. Thus far, findings from adult populations have demonstrated a significant link between the domains of Disinhibition, Detachment, and Psychoticism, and problematic gambling behavior [44,46]. Moreover, the link between personality traits and over-indebtedness is not well understood. Based on these considerations, we hypothesize that.

• H1: Disinhibition, Detachment and Psychoticism are significantly related to problem gambling.
• H2: Disinhibition, Detachment and Psychoticism are significantly related to over-indebtedness.

To our knowledge, this is the first study to examine the relationship between maladaptive personality traits and over-indebtedness among problem gamblers. By exploring this link, our study contributes to the broader literature on the negative outcomes of problem gambling and sheds light on the factors that may pose a risk of over-indebtedness among problem gamblers. Additionally, applying a dimensional and clinical personality model can help elucidate the exact maladaptive personality traits that are specific to problem gambling, and thus reconceptualize diagnostic means by drifting away from symptom counting.

Past research demonstrates that gender moderates the relationship between personality and problem gambling [25] and motivation to gamble [4]. For instance, the relationship between Openness and problem gambling has been found to be significantly negative for males but non-significantly positive for females [25]. Considering the recently reported gender differences in Sweden regarding problem gambling [17,18], and that gender differences in personality are larger in more gender-equal countries [[55], [56], [57], [58]], this study explores gender as a potential moderator in the relationships of maladaptive personality traits towards problem gambling and over-indebtedness.

Problematic gambling often co-occurs with alcohol [59] and substance use problems [60,61], therefore we controlled for both alcohol and drug use problems.

## 2 Methods

The present study is a cross-sectional survey study carried out in the period between April 28th and May 9th^,^ 2022, conducted on a sample of adults who gamble. Participants were recruited from the web panel of the market survey company Ipsos, which typically addresses individuals that sign up on their web panel for different surveys and polls. Partaking in the study was voluntary and compensated through Ipsos’ internal system with credits corresponding to around 1 Euro, with which participants can purchase items from a range of goods. The study was approved by the Swedish Ethical Review Authority (file number: 2022-01332-01).

### 2.1 Participants

The aim was to address roughly 1000 adult individuals, based on previous research in the field [18]. A sample of 1479 Swedish adults who gamble, was recruited. In order to participate in the survey, individuals had to be 18 years and above, and to have gambled at least once to four times in the last 12 months. Participants with less severe gambling habits were included to best observe personality differences. Before the survey, participants were asked to sign an informed consent form. Characteristics of the sample are displayed in Table 1.

**Table 1:** Sample characteristics (N = 1479).

	n	%
Gender		
- Male	966	65
- Female	513	35
Age		
- 18-24	80	6
- 25-29	153	10
- 30-39	343	23
- 40-49	255	17
- 50-59	263	18
- 60-69	258	17
- 70+	127	9
Living conditions		
- Alone with children	89	6
- Alone without children	379	26
- With partner and children	466	31
- With partner, without children	502	34
- With parents	43	3
Main occupation		
- Student	84	6
- Working	1039	70
- Job seeking	47	3
- Retired	40	3
- Laid off	269	18
Highest level of education		
- Primary school	92	6
- High school	625	42
- University degree	501	34
- University studies without degree	229	15
- Other	32	2
Over-indebtedness reported		
- Yes	91	6
- No	1388	94
Past-year gambling activities reported		
- Online casino	548	37
- Land-based casino	154	10
- Online horse betting	755	51
- Land-based horse betting	305	21
- Live sports betting	680	46
- Non-live sports betting	745	50
- Online poker	278	19
- Land-based poker	184	12
- Land-based slot machines	227	15
- Online bingo	330	22
- Microtransactions	207	14
Ever felt a need to seek treatment for alcohol problems		
- Yes	114	8
- No	1356	92
Ever felt a need to seek treatment for drugs problems		
- Yes	61	4
- No	1409	96

### 2.2 Measures

#### 2.2.1 Socio-demographic data

Participants were asked demographic questions and questions about seeking treatment due to alcohol and drug problems. The survey included questions about a range of games, both land-based and online, such as casino games, horse betting, sports betting, poker and slot machines, online bingo, and in-game gambling (microtransactions). Participants were asked whether they have played these types of games in the last 12 months (Table 1).

#### 2.2.2 Problem gambling

Problem gambling was measured using the Problem Gambling Severity Index (PGSI) [62], which has been widely used in Swedish studies [63,64]. PGSI is a 9-item scale, with four of its items measuring problem gambling behaviors (betting, tolerance, chase, borrowing), and the remaining five items assessing the unpleasant results of gambling (felt problem, being criticized, felt guilty, health problem, financial problem). Participants answer each question on a scale from 0 to 3 (0 = never, 1 = sometimes, 2 = most of the time, 3 = almost always). According to the PGSI scoring system, participants are classified into four categories: no-risk gamblers (score of 0), low-risk gamblers (score of 1–2), moderate-risk gamblers (score of 3–7), and problem gamblers (score of 8 or more) [63]. In this study, PGSI's internal consistency was proven to be excellent (α = 0.95).

#### 2.2.3 Maladaptive personality

The Personality Inventory for DSM-5 from the latest version of the Diagnostic and Statistical Manual of Mental Disorders (DSM-5) [38] assesses five personality trait domains: Negative Affectivity, Detachment, Antagonism, Disinhibition and Psychoticism. Originally, the PID-5 comprises 220 items, but recent studies have supported the validity of a short version (PID-5-BF) [40,65,66]. This study utilized the Swedish version of the PID-5-BF, which includes 25 items on a 4-point Likert scale from 0 (very false or often false) to 3 (very true or often true) [40]. In the present study, the overall scale exhibited excellent internal consistency (α = 0.91), with Cronbach's Alphas being acceptable for Disinhibition (α = 0.79), Detachment (α = 0.74), Psychoticism (α = 0.79), Negative Affectivity (α = 0.77) and Antagonism (α = 0.74).

#### 2.2.4 Over-indebtedness

In order to identify over-indebtedness in the sample, participants were presented with the following question: “During the past year, have you taken a loan primarily to be able to gamble or to pay debts that have been caused by gambling?”. The answer “yes” is indicative of over-indebtedness. This study made use of the subjective definition of over-indebtedness, which is based on the participants’ self-reporting of financial debts [67]. The subjective definition has been widely used in research settings in Sweden [17,18,68], as well as in reports published by the [69].

#### 2.2.5 Statistical analysis

Data analyses were conducted using jamovi version 2.0.0.0. All variables were initially screened in RStudio for missing data, distribution abnormalities, and outliers. The proportion of missing data was 0.24%, and to handle missing data, listwise deletion was performed since the results of the Missing Completely at Random (MCAR) test yielded non-significant results (*p* = .99). Ten participants appeared as outliers in the dataset as their z-scores were above 3.29, considering the large sample size. Testing was performed with and without the outliers, as far as the independent variables are concerned. Subsequently, these were not removed as they did not influence the results significantly.

Pearson correlation coefficients were calculated to examine the relationships among the study variables (Table 2), and Chi-square values were computed for categorical data. Given the multiple correlations performed, we applied Bonferroni's correction to control for the false discovery rate with regard to the independent and dependent variables. The adjusted p-value threshold for statistical significance was set at p < .005, and the correlations between the PID-5-BF variables and the dependent variables (PGSI and over-indebtedness) remained significant (p < .001). Generalized linear models (GLMs) taking into consideration the data's overdispersion were conducted for all other estimations. For the hypotheses involving PGSI as the outcome variable, Poisson loglinear models were tested assuming a quasi-Poisson distribution and using the log link function [70]. This decision was made because the normality assumption was violated (p < .001), and the variance was larger than the mean (*M* = 2.4, *SD* = 4.9, σ^2^ = 23.5). The outcome variable's skewness was found to be 2.62, indicating a right-skewed distribution, and the kurtosis was found to be 6.86, indicating a more heavy-tailed compared to the normal distribution.

**Table 2:** Pearson's correlation coefficients for study variables.

Variable	1	2	3	4	5	6	7	8
Gender	–							
PID-5-BF^a^								
1. Negative Affectivity	.27***	–						
2. Detachment	.06*	.49***	–					
3. Antagonism	−.03	.42***	.38***	–				
4. Disinhibition	.09***	.57***	.47***	.59***	–			
5. Psychoticism	.11***	.62***	.54***	.56***	.68***	–		
PGSI^b^	.10***	.36***	.28***	.47***	.51***	.46***	–	
Over-indebtedness	.07**	.16***	.14***	.35***	.32***	.26***	.64***	–

*p < .05, **p < .01, ***p < .001.

a Personality Inventory for DSM-5-Brief Form.

b Problem Gambling Severity Index.

Given the high correlation between PID-5-BF subscales run prior to the analyses (Table 2), each independent variable of the PID-5-BF was initially tested in a univariate analysis. A model including all five independent variables was then computed to observe any change in the relationship with the outcome variable. For the hypotheses involving the categorical variable of over-indebtedness as the outcome, logistic regression models were computed. At the final step of the analyses, the control variables of drug and alcohol problems were inserted in the models. GLMs were also used to test the interactions of gender with the PID-5-BF traits. The independent variables were standardized prior to the analyses and the factor of gender was coded with 0 and 1 (0 = males, 1 = females).

## 3 Results

### 3.1 General characteristics of the sample

Twelve percent of the participants met the criteria for problem gambling, and another 12% met the criteria for moderate-risk gambling. Another 18% met the criteria for low-risk gambling, whereas the remaining 58% had no problem gambling. Problem gambling was significantly more common in women (16.2%) than in men (9.4%), and the percentage of moderate-risk gambling was 12.7% in women and 11.7% in men, χ^2^(3, *N* = 1479) = 15.9, p < .001. With regards to over-indebtedness, 8.6% of female participants and 4.9% of male participants reported over-indebtedness, χ^2^(1, *N* = 1479) = 7.99, p = .005. In total, 6% of all participants reported over-indebtedness.

### 3.2 Gender comparison of sociodemographic and other parameters

Men were more likely to be 50 years old and above, while women were more likely to be between 30 and 49 years of age, χ^2^(6, *N* = 1479) = 35.9, *p* < .001. Men were more likely to be unemployed and women were more likely to have retired from work or to be job seekers, χ^2^(4, *N* = 1479) = 16.2, *p* = .003. With regards to monthly income, men generally reported more earnings than women, χ^2^(9, *N* = 1479) = 51.7, *p* < .001. While the two gender groups were not significantly different with regards to seeking therapy for alcohol dependence χ^2^(1, *N* = 1479) = 1.40, *p* = .23, seeking therapy for drug dependence was significantly more prevalent in women χ^2^(1, *N* = 1479) = 29.9, *p* < .001, as well as having received therapy for a mental illness χ^2^(1, *N* = 1479) = 98.5, *p* < .001.

In terms of gambling activities, women were significantly more likely to report using online casino χ^2^(1, *N* = 1479) = 42.9, *p* < .001, land-based casino χ^2^(1, *N* = 1479) = 8.80, *p* = .003, slot machines χ^2^(1, *N* = 1479) = 12.4, *p* < .001, online bingo χ^2^(1, *N* = 1479) = 74, *p* < .001, and microtransactions χ^2^(1, *N* = 1479) = 4.32, *p* = .03. Men were significantly more likely to report using both live sports betting χ^2^(1, *N* = 1479) = 52.1, *p* < .001, and non-live sports betting χ^2^(1, *N* = 1479) = 130, *p* < .001.

### 3.3 Associations of the PID-5-BF on problem gambling and over-indebtedness

To evaluate the first hypothesis testing whether the PID-5-BF domains of Disinhibition, Detachment and Psychoticism were significantly related to problem gambling, quasi-Poisson loglinear models were estimated. At first, all five trait domains were tested in separate analyses to examine their unique contributions to the outcome variable. All five variables were associated to problem gambling, with Disinhibition showing the largest effect size *b* = 0.71, *OR* = 2.04, 95% CI [1.92, 2.17], *p* < .001, and Detachment showing the smallest effect size *b* = 0.51, *OR* = 1.68, 95% CI [1.54, 1.83], *p* < .001. The results of the final model which included all five trait variables partially supported the first hypothesis, as all of them were uniquely associated with problem gambling, except for Detachment, even after adding the control variables to the model (Table 3).

**Table 3:** Quasi-Poisson loglinear analyses with PGSI^a^ as dependent variable before and after inserting control variables.

				95% (*OR*) CI			
Variable	*b*	*SE*	*OR*	*LL*	*UL*	z	*p*
Step 1							
Intercept	0.514	.052	1.67	1.50	1.85	9.82	<.001
Negative Affectivity	0.144	.054	1.16	1.03	1.28	2.66	.008
Detachment	0.025	.050	1.03	0.93	1.13	.503	.615
Antagonism	0.237	.041	1.27	1.17	1.38	5.73	<.001
Disinhibition	0.342	.052	1.41	1.27	1.56	6.50	<.001
Psychoticism	0.166	.056	1.18	1.05	1.32	2.93	.003
Step 2							
Intercept	0.771	.078	2.16	1.85	2.51	9.88	<.001
Negative Affectivity	0.161	.054	1.18	1.05	1.31	1.99	.003
Detachment	0.014	.050	1.01	0.92	1.12	.283	.777
Antagonism	0.210	.041	1.23	1.14	1.34	5.08	<.001
Disinhibition	0.322	.053	1.38	1.24	1.53	6.07	<.001
Psychoticism	0.124	.057	1.13	1.01	1.27	2.17	.030
Alcohol problems^b^	0.160	.124	1.17	0.92	1.49	1.29	.197
Drug problems^b^	0.436	.136	1.55	1.18	2.02	3.21	.001

*Note. N* = 1479. McFadden's *R*^2^ = 0.36. *OR* = odds ratio; CI = confidence interval; *LL* = lower limit; *UL* = upper limit.

a Problem Gambling Severity Index.^b^0 = no, 1 = yes.

Logistic regression was used to investigate the second hypothesis testing whether the PID-5-BF domains of Disinhibition, Detachment and Psychoticism were significantly related to over-indebtedness. At first, all five trait domains were tested in separate analyses to examine their unique contributions to the outcome variable. All five variables showed significant results, with Antagonism showing the largest effect size *b* = 1.07, *OR* = 2.90, 95% CI [2.41, 3.52], *p* < .001, and Detachment showing the smallest effect size *b* = 0.57, *OR* = 1.77, 95% CI [1.45, 2.17], *p* < .001. In the final model which included all five trait variables, the hypothesis was partially supported, as only Disinhibition and Antagonism were uniquely associated to over-indebtedness (Table 4).

**Table 4:** Logistic regression analyses with over-indebtedness^a^ as dependent variable before and after inserting control variables.

				95% (*OR*) CI			
Variable	*b*	*SE*	*OR*	*LL*	*UL*	z	*p*
Step 1							
Intercept	−3.420	.166	0.03	0.02	0.04	−20.6	<.001
Negative Affectivity	−0.180	.166	0.83	0.60	1.15	−1.07	.280
Detachment	−0.109	.157	0.90	0.65	1.21	−0.69	.488
Antagonism	0.747	.129	2.11	1.64	2.73	5.80	<.001
Disinhibition	0.624	.155	1.87	1.38	2.54	4.02	<.001
Psychoticism	0.118	.172	1.12	0.80	1.58	0.69	.493
Step 2							
Intercept	−2.263	.228	0.10	0.07	0.16	−9.94	<.001
Negative Affectivity	−0.139	.185	0.87	0.60	1.24	−0.75	.454
Detachment	−0.144	.176	0.87	0.61	1.21	−0.82	.413
Antagonism	0.694	.143	2.00	1.52	2.66	4.86	<.001
Disinhibition	0.543	.176	1.72	1.22	2.43	3.09	.002
Psychoticism	−0.041	.194	0.96	0.65	1.40	−0.21	.831
Alcohol problems^a^	1.433	.319	4.19	2.21	7.75	4.49	<.001
Drug problems^a^	1.641	.387	5.16	2.40	10.9	4.24	<.001

*Note. N* = 1479. McFadden's *R*^2^ = 0.33. *OR* = odds ratio; CI = confidence interval; *LL* = lower limit; *UL* = upper limit.

a 0 = no, 1 = yes.

### 3.4 Moderation of gender

To test the interactions between gender and Disinhibition, Detachment and Psychoticism, respectively, the main and interaction effect was added to the GLM regressions with problem gambling and over-indebtedness as dependent variables. Moderation was observed when using problem gambling as the dependent variable, but significant interactions with gender were reported only for Negative Affectivity *b* = 0.24, *OR* = 1.28, 95% CI [1.07, 1.53], *p* = .0037, Disinhibition *b* = 0.18, *OR* = 1.20, 95% CI [1.07, 1.36], *p* = .003, and Psychoticism *b* = 0.18, *OR* = 1.20, 95% CI [1.05, 1.38], *p* = .008. An examination of the simple slopes revealed that the slopes were significantly more positive for men, with regards to Disinhibition χ^2^(1, *N* = 1479) = 335, *p* < .001, Psychoticism χ^2^(1, *N* = 1479) = 250, *p* < .001 and Negative Affectivity χ^2^(1, *N* = 1479) = 147, *p* < .001. Males with high levels on these subscales were more likely to report problem gambling, while females with low levels on these subscales were more likely to report problem gambling.

Gender did not moderate the separate relationships between the PID-5-BF domains and over-indebtedness. This was tested by inserting the main and interaction effects into the logistic regression models. The results of these analyses were all non-significant.

## 4 Discussion

To our knowledge, this is the first study to investigate the relationship between the PID-5 trait domains and over-indebtedness, as well as problem gambling, in a sample of gamblers while controlling for alcohol and drug use problems. Almost all trait domains were uniquely related to problem gambling, while only Disinhibition and Antagonism were uniquely related to over-indebtedness. According to the available literature, this is also the first study to investigate the moderating role of gender in the explored relationships. The separate relationships of Negative Affectivity, Disinhibition and Psychoticism with problem gambling were significantly more positive for men than women, while problem gambling and over-indebtedness were more prevalent in females.

Unlike studies linking GD to Detachment [45,46], the latter did not show a unique relation to problem gambling, while Antagonism and Disinhibition showed the strongest associations to problem gambling. Research indicates that a high level of Antagonism is more prevalent among high-risk gamblers compared to low-risk gamblers [45]. Gambling severity has also been connected to a low level of Agreeableness; a factor reversely related to Antagonism [[22], [23], [24], [25]]. In the clinical context, Antagonism in pathological gamblers may indicate propensities toward asocial behavior [71,72], and it could emphasize the high comorbidity between gambling and antisocial personality disorder [73].

The findings relating Disinhibition to problem gambling are in accordance with many studies that have supported the presence of impulsivity in problematic gambling [27,28,31,74]. Indeed, what helps differentiate best between healthy controls and disordered gamblers, is the higher scores of the latter on the PID-5 domain of Disinhibition [46]. Considering the existing evidence on the relation between impulsivity and gambling severity [26,75,76], this result was expected and is further supported by the previous classification of GD as an impulse-control disorder in DSM-IV [77].

As hypothesized, Psychoticism and problem gambling were related, which agrees with findings on the relationship between Psychoticism and chasing losses [44] and GD [46]. Other studies have reported that individuals with GD exhibit higher levels of psychoticism [20,21], however, it is critical to recognize that these studies did not make use of the PID-5 model. Instead, psychoticism has been previously explored through impulsivity subscales, which have been found to reliably distinguish a specific type of impulsivity, one that correlates highly with the personality trait of psychoticism [78]. This is a key component in understanding the present results, especially since Psychoticism and Disinhibition were both related to PG, as well as shared the strongest correlation among all PID-5 domains (Table 2). Though the underlying mechanisms linking Psychoticism and PG are still unidentified, it could be hypothesized that individuals with higher levels of Psychoticism may be gambling as a way to cope with psychological distress. Moreover, people with problematic gambling behavior may experience strange beliefs and cognitive distortions that can enhance their gambling involvement [[79], [80], [81], [82]].

Regarding our second hypothesis, only Disinhibition and Antagonism were related to over-indebtedness. While the role of Antagonism is still unexplored, some findings could support the role of Disinhibition. A relationship between impulsivity and financial debts has been reported in non-clinical samples [53], and more studies suggest that impulsive individuals with low self-control have a higher propensity to over-indebtedness specifically [[83], [84], [85]]. The present study however is the first one to examine personality and over-indebtedness in a population that includes problem gamblers, considering specifically the PID-5 model. While most associations presented cannot be directly supported by the known literature, our findings can act as a turning point in psychiatric research, where measures of maladaptive personality traits could be utilized in studying over-indebtedness.

Although males were the majority among gamblers, females reported higher rates of both moderate gambling and problem gambling, as well as higher levels of over-indebtedness. While this is atypical to what a variety of reports suggest globally [[3], [4], [5], [6], [7], [8], [9],^86^], the present results are in agreement with the most recent Swedish studies [17,18,63] as well as one study from the USA [87]. In accordance also to recent Swedish research indicating gender differences in preferred gambling modalities [8,17], in this study men demonstrated higher rates of both live and non-live sports betting, while women demonstrated higher rates of online casino and online bingo. Interestingly, online casino is the most common gambling type being advertised on Swedish media, where ads encourage females to play by promoting its ease of usability [88]. Finally, the present results could also be explained, by the telescoping effect, a phenomenon where women tend to experience an escalation to gambling problems and a shorter time between initiation of gambling and the onset of problem gambling symptoms, compared to men [89]. Nonetheless, the complexity and possible joint contribution of various factors, along with the current state of online gambling exposure in Sweden, should be further examined with a focus on individual differences in problem gamblers.

Gender also moderated the relationships of Negative Affectivity, Disinhibition and Psychoticism with problem gambling, in the way that men with high levels in these domains were more likely to report problem gambling, compared to women. Even though the relationships were significant, they should be observed with caution, as the effect sizes were small, indicating a weak practical significance. Since past research on the PID-5 model has refrained from drawing conclusions on gender differences due to the low rate of female participants [47], it is important to consider the present results as exploratory information that can help in the formulation of stronger hypotheses and research methods.

### 4.1 Limitations

Aside from its strong properties, including the large sample, this study comes with some limitations. First, although valuable insights are provided, the cross-sectional nature of the study design does not allow for establishing causality in the observed relationships. Secondly, the data collection method through a web survey may have posed some risks such as recall bias and bias caused by the selection of participants through a web panel. The participants of this study have voluntarily chosen to be part of Ipsos’ web panel as potential survey respondents. Other limitations are the self-report methods used that could pose a risk of social desirability, and the exploration of important risk factors through brief questions instead of separate instruments. One such question was the item evaluating the presence of over-indebtedness, which includes two different aspects: borrowing money to gamble and borrowing money to pay off debts caused by gambling. Future research should explore more refined methods of exploring over-indebtedness, in ways that distinguish the reasons for borrowing money, but also account for the size of the amounts being borrowed.

The choice of the PID-5 model was justified, considering that it has demonstrated satisfactory convergent validity with clinical interviews [90], and it has also been validated in a common community sample [40]. Nonetheless, the notion that maladaptive traits load onto one general psychopathological factor, the p-factor [91], should be taken into consideration. Indeed, in the present analysis, all PID-5-BF domains were highly correlated.

Even though the present results were derived from a population of gamblers, the generalizability of results is limited to the Swedish context. There is a high need for more studies on problem gambling and over-indebtedness in other European countries and worldwide, in order to elucidate the role of maladaptive personality traits and gender differences, which could manifest in different ways in other cultural contexts.

Lastly, it is of high importance for future studies to determine the robustness of the observed and non-observed interactions through replication and possibly assess whether the connections of personality with over-indebtedness and problem gambling are reliant on more factors, such as gambling modality, psychiatric comorbidity, and treatment status. In this regard, researchers should prioritize the collection of diverse samples allowing for sufficient variability and greater statistical power in order to detect and examine the role of these factors.

## 5 Conclusion

The present study demonstrates that, after controlling for alcohol and drug use problems, Disinhibition and Antagonism were found to have the strongest associations with problem gambling, as well as that only these domains were uniquely related to over-indebtedness. The fact that problem gambling and over-indebtedness were more common among women highlights the necessity to further explore gender and individual differences, in Sweden and globally. Knowledge about the role of maladaptive personality could be proven beneficial in clinical settings, where it could play a pivotal role in early diagnosis and intervention. Since problem gambling can constitute a diagnostic challenge at early stages, it is important to employ models that point towards pathological personality traits and differentiate elements that could denote problematic versus no-risk gambling behaviors.

## Author contribution statement

Nikoleta Komzia: Conceived and designed the experiments; Performed the experiments; Analyzed and interpreted the data; Wrote the paper.

Martin Bäckström: Analyzed and interpreted the data.

Anders Håkansson: Performed the experiments; Contributed reagents, materials, analysis tools or data.

## Data availability statement

Data will be made available on request.

## Declaration of competing interest

The authors report no conflicts of interest related to the present research project.

Dr. Anders Håkansson has a position as a researcher at Lund University which is sponsored by the state-owned gambling operator of Sweden, and he also has research funding from the research councils of the state-owned gambling operator, Svenska Spel AB, and the state-owned alcohol monopoly. None of these organizations had any role in - and no influence on - the present work.
